# Podiatry in continence

**DOI:** 10.1186/1757-1146-4-S1-P18

**Published:** 2011-05-20

**Authors:** Kelly Edwards, Annette Davis

**Affiliations:** 1 Podiatry Allied Health Assistant, Rehabilitation and Aged Care Services, Kingston Centre, Cheltenham, 3192, Victoria, Australia; 2 Podiatry Manager, Rehabilitation and Aged Care Services, Kingston Centre, Cheltenham, 3192, Victoria, Australia

## 

Continence has been implicated as a contributing factor in falls related injury. In aged care rehabilitation, continence was identified by podiatry as an issue in regards to footwear selection and was an opportunity to improve falls prevention education and improved quality of life for the patient. The key elements of this education focused on the importance of slip-resistance in footwear and washability. Kingston Centre is an aged care rehabilitation service that has an existing podiatry lead footwear prescription service, where patients can purchase footwear with optimal features for a reduced cost. Most of the footwear is machine-washable and several have an outsole configuration that actively grips the floor in stance, which is advantageous on a wet surface. An education package was developed for continence nurses to improve knowledge of footwear, and foot care issues and promote referral to podiatry. Up-skilling continence nurses in the importance of footwear selection, footwear laundering as well as general foot care, has improved awareness of the relationship between appropriate footwear, falls prevention and continence. Anecdotally, there has been a reduction in falls in relation to continence issues. This will be further investigated in future. Of particular note is that the delivery of educative in-services and the majority of footwear prescription and fitting is performed by a skilled podiatry allied health assistant. The podiatrist was in engaged in the development but has a now assumed a smaller consultative role. Engaging a podiatry allied health assistant in this way has expanded the service while still allowing the professional staff to focus high risk patients. It also has improved quality of life implications for a population that would not have been seen by podiatry.

